# Evaluation of antiarrhythmia drug through QSPR modeling and multi criteria decision analysis

**DOI:** 10.1038/s41598-025-14892-2

**Published:** 2025-08-09

**Authors:** Shereen Iqbal, Hifza Iqbal, Muhammad Akhtar Tarar, Muhammad Farhan Hanif, Osman Abubakar Fiidow

**Affiliations:** 1 Department of Mathematics and Statistics, The University of Lahore, Defence Road Campus, Lahore, Pakistan; 2 Department of Civil Engineering, The University of Lahore, Defence Road Campus, Lahore, Pakistan; 3 Department of Public Health, Faculty of Health Science, Salaam University, Mogadishu, Somalia

**Keywords:** Correlation coefficient, Heatmap, QSPR analysis, Topological indices, MCDM, TOPSIS, SAW, Chemistry, Mathematics and computing

## Abstract

This study explores how topological indices (TIs), which are mathematical descriptors of a drug’s molecular structure, can support to predict vital properties and biological activities. This understanding is a key for more effective drug design. We focused on drugs used to treat several arrhythmia conditions, including tachycardias, bradycardias, and premature beats. Our approach combines molecular modeling with decision-making techniques to offer a cost-effective way to understand how these drug molecules behave. Our procedure started with calculating topological indices for the chemical structures of these medications to extract information about their features. We then established quantitative structure-property relationship (QSPR) models using quadratic regression, training and validating them. We concentrated on TIs that showed a strong correlation$(> 0.7)$ with physicochemical properties. Each property was also weighted, based on its correlation with the topological indices. As a final point, to aid in informed decision-making, we employed multiple-criteria decision-making approaches Technique for Order Preference by Similarity to Ideal Solution TOPSIS and Simple Additive Weighting SAW to rank the anti- arrhythmia medications. Drug Amiodarone ranked highest due to strong correlation with boiling point and polarizability. The study also highlights the potential of machine learning to analyze large datasets, allowing for accurate predictions of chemical behavior. This comprehensive method can facilitate the detection of new drugs with valuable qualities and improve our understanding of how chemical structures affect drug effectiveness.

## Introduction

An arrhythmia is a condition characterized by an irregular heartbeat, meaning the heartbeats either occur too quickly (tachycardia), too slowly (bradycardia), or unpredictably. This abnormality can delay normal blood flow, possibly leading to severe health problems such as stroke, heart failure, or unexpected cardiac arrest. Arrhythmias can stem from several factors, including structural heart abnormalities, electrolyte imbalances, and underlying medical conditions. A systematic understanding of how arrhythmias develop and their possible repercussions is vital for developing effective cures^1,2^.

**Fig. 1:**
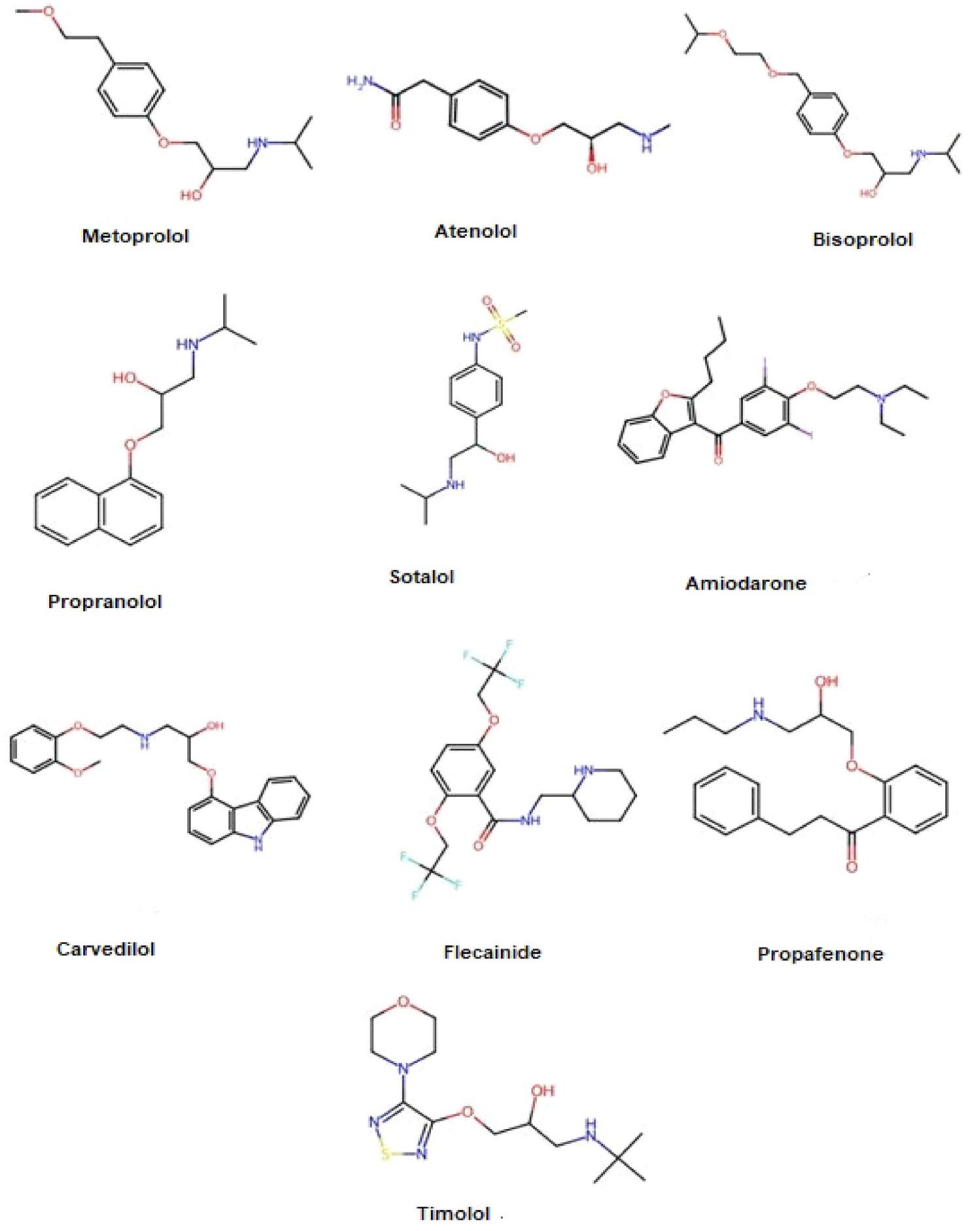
Structures of anti-arrhythmia drugs.

Our research included ten effective medications for treating arrhythmias, as illustrated in Fig. 1 and sourced from existing literature. These include Beta-blockers such as Metoprolol^3^, Atenolol^4^, Bisoprolol^5^, and Propranolol^6^ work by slowing heart rate and reducing myocardial oxygen demand. Timolol^7^ is another beta-blocker used for heart rate control in specific arrhythmias. Zhang et al.^13^ presented that exosomal derived from M2 macrophages inhibits CVB3-induced viral myocarditis by regulating PKM2/HIF-1a, which results in macrophages metabolism, and immunoreaction. Dual-action agents such as Sotalol^8^ acts as both a beta-blocker and an anti arrhythmic, helping to maintain sinus rhythm.

Li et al.^14^ developed an upper-tyramine (TY) signal amplification probe based on nanozyme for myocarditis-related miRNAs with high sensitivity in heart without pre-amplification, and established a nanospring TMB microplate method for a rapid clinical diagnostic of myocarditis. Broad-spectrum anti arrhythmics are Amiodarone^9^ and Carvedilol^10^ are commonly prescribed for more severe arrhythmias due to their ability to control heart rate. Pei et al.^15^ designed extracellular vesicles for multitargeted immunomodulatory therapy, which could provide a novel approach to treat viral myocarditis based on enhancing host immune regulation.

Flecainide^11^, and Propafenone^12^ are Sodium channel blockers that stabilize electrical signals of heart. These drugs were chosen due to their well-known mechanisms of action that effectively target various types of arrhythmias. While generally effective, some have minor side effects, such as fatigue and dizziness with metoprolol and atenolol. More severe adverse effects associated with amiodarone are thyroid dysfunction, pulmonary toxicity, and sotalol has the risk of inducing arrhythmias by prolonging the QT interval. Zhang et al.^16^ published the first case of subcutaneous ICD placement in a child with Timothy syndrome demonstrating a new technique for pediatric patient management of cardiac risk. Wei et al.^17^ performed a systematic review and meta-analysis that indicated that Shenfu injection was effective and safe in the treatment of bradyarrhythmia, supporting that it can be applied as a complementary therapy. Liu et al.^18^ in mice, they had different effects on cardiac function and thus can be new targets for heart failure therapies. To accelerate the development of more effective drugs, in our research, by applying topological indices, entropy, and concepts from chemical graph theory, we were able to forecast the physicochemical properties of these substances.

In mathematics, a graph is a fundamental structure consisting of a set of vertices (or nodes) and a set of edges (or links) that connect pairs of these vertices. Formally, a graph *G* is an ordered pair (V, E), where V is the set of vertices and E is the set of edges. Graph theory is the branch of mathematics that studies graphs. It explores the properties, structures, and algorithms related to graphs^19^. Early contributions to graph theory can be traced back to Leonhard Euler’s work on the Koinigsberg bridge problem in 1736, which is often considered the birth of the field^20^. Since then, graph theory has grown into a vast and vigorous area of research with many uses.

Chemical graph theory is a particular area that applies graph theory to model and recognize chemical structures. In this context, atoms are classically symbolized as vertices, and the chemical bonds between them are represented as edges. This permits chemists to define molecular structures using graph-theoretical conceptions^21^. By converting molecules into graphs, various topological indices can be calculated to predict physicochemical properties, biological activities, and reactivity of chemical compounds. Innovative work in chemical graph theory by researchers like Wiener and Hosoya laid the basis for its improvement^22,23^. It has turned into an essential tool in cheminformatics, drug innovation, and materials science.

In QSPR analysis, graph-based structures play a crucial role in developing innovative drugs^24^. We have established relationships between molecular structure and physicochemical properties by calculating six specific indices. The findings contribute to the growing literature on topological indices and their applications in pharmaceutical research.

Huang et al.^25^ employed XGBoost and regressive models in the QSPR studies of glaucoma drugs, in successful relation of topological descriptors and pharmacological activities for the predictive purposes. Qin et al.^26^, mined Python based topological modeling to straw test pulmonary cancer drugs and accomplished 821 reliable QPSR predictions via integration of graph-theoretic indices and computation. Qin et al.^27^ investigated anti-arrhythmic drug features utilizing Python and topological indices, an indicative high accuracy of QSPR-based physicochemical property predicition has been demonstrated. Wei et al.^28^ for several drugs by the linear regression models, which proved that QSPR could be a good alternative to estimate physical properties of compounds from mathematical descriptors.

Researchers have created various graph polynomials to make calculation of topological indices easier. For example, the Hosoya polynomial^29^ simplifies indices based on distance, the M-polynomial helps with calculations based on vertex degrees^30^, and the neighborhood M-polynomial handle indices related to the sum of degrees of neighboring vertices. These methods have been widely applied to many different molecules, graph networks, and structures in various studies. In 2021, Mondal et al.^31^ introduced the neighborhood M-polynomial. This tool streamlines the calculation of neighborhood degree sum-based indices, much like how the M-polynomial simplifies degree-based index computations. Essentially, it allows the direct and easy computation of these indices. The neighborhood M-polynomial of a graph $\varGamma$, denoted by $NM(\varGamma ;s,t)$, is defined as:

1 $$\begin{aligned} NM(\varGamma ;s,t) = \sum _{k \le l} m_{kl}(\varGamma ) s^k t^l \end{aligned}$$

where $m_{kl}(\varGamma )$, is the number of edges *uv* in $\varGamma$ such that $d_u = k$ and $d_v = l$, where $d_u$ and $d_v$ are the degrees of vertices *u* and *v*, respectively. Table 1 summarizes the relationships between neighborhood degree sum-based topological indices, and their corresponding NM-polynomial.

**Table 1:** Degree-based topological indices derivations.

Topological indices	Derivation from*NM*(*s*, *t*)
$M_1$	$(\Game _{s}+\Game _{t})(NM(\varGamma ))|_{s=t=1}$
$M_2$	$(\Game _{s}\Game _{t})(NM(\varGamma ))|_{s=t=1}$
$NM_1$	$(\delta _{s}+\delta _{t})(NM(\varGamma ))|_{s=t=1}$
$NM_2$	$(\delta _{s}\delta _{t})(NM(\varGamma ))|_{s=t=1}$
*NH*	$(2S_{s}J)(NM(\varGamma ))|_{s=t=1}$
*NSS*	$\sum _{rs \in E(\varGamma )} \sqrt{\frac{\Game _s \Game _t}{\Game _s + \Game _t}}(NM(\varGamma ))_{s=t=1}$

Where, $$\Game _{s}= s\frac{\partial g}{\partial s}$$, $$\Game _{t} = t\frac{\partial g}{\partial t}$$, $$S_{s}=\int _{0}^{s}\frac{g(l,t)}{l}dl$$, $$S_{t}=\int _{0}^{t}\frac{g(s,l)}{l}dl$$, $$J(g(s,t))=g(s,s)$$,

$$\Game ^{1/2}_s=\sqrt{s\frac{\partial g}{\partial s}}\sqrt{g(s,t)}$$, $$\Game ^{1/2}_t=\sqrt{t\frac{\partial g}{\partial t}}\sqrt{g(s,t)}$$

$$S^{1/2}_{s}=\int _{0}^{s}\sqrt{\frac{g}{l}}dl\sqrt{g(s,t)}$$, $$S^{1/2}_{t}=\int _{0}^{t}\sqrt{\frac{g}{l}}dl\sqrt{g(s,t)}$$ are operators.

This motivation behind this study is to introduce a novel, cost-effective method to predict vital properties and biological activities of anti-arrhythmia drugs by combining molecular modeling with decision-making techniques and utilizing topological indices. The study claims that this model is better than the existing ones by offering a rapid approach to predict drug properties and rank alternatives, which complements traditional time-consuming and expensive clinical trials.

## Methodology

**Fig. 2:**
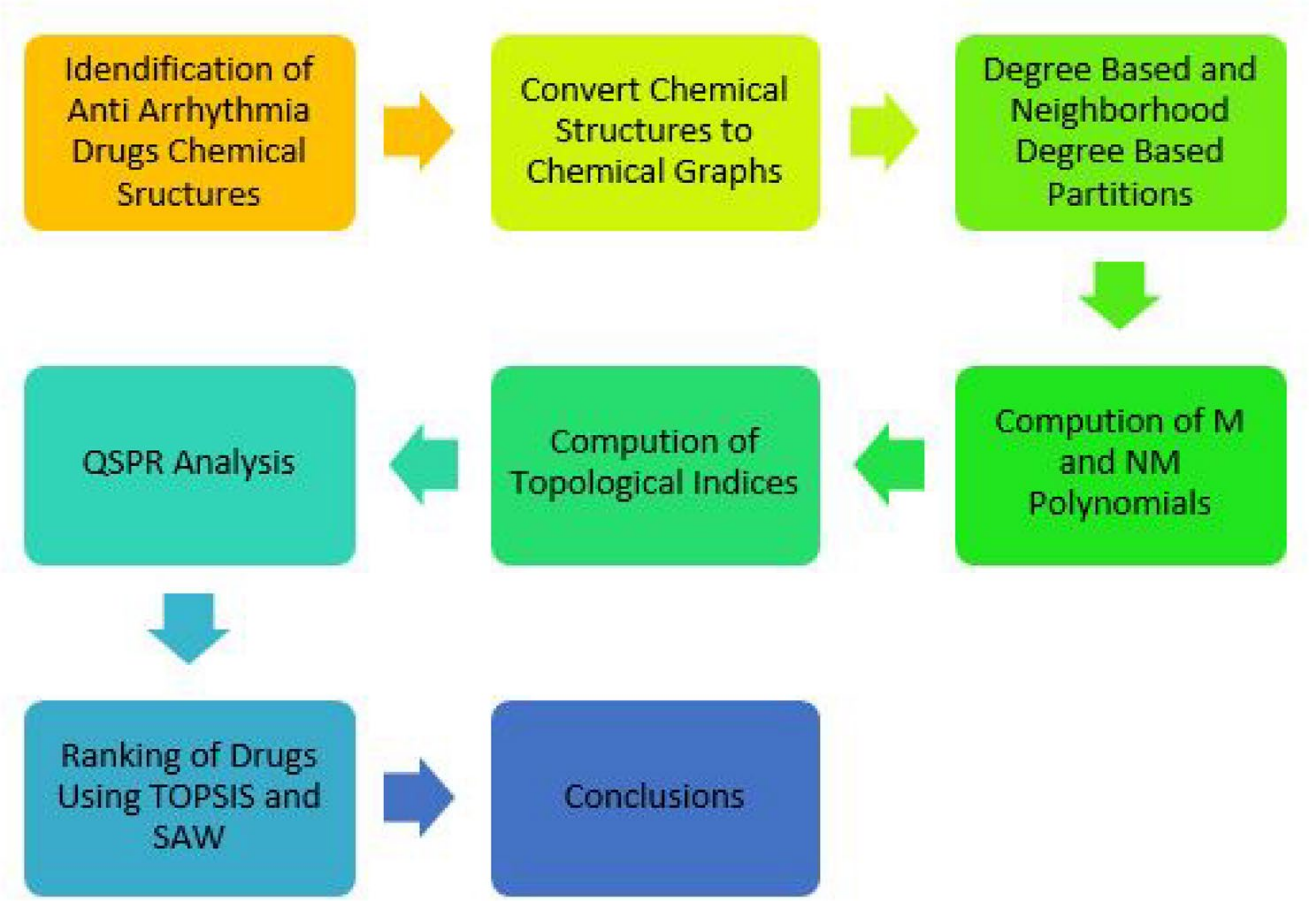
Methodology.

This study performed a QSPR (Quantitative Structure-Property Relationship) analysis to rank medicines for arrhythmia treatment Fig. 1. We investigated how topological indices relate to key drug properties that we obtained from Camspider like boiling point (BP), molar volume (MV), density (D), polarizability (P), BFC, KOC and flash point (FP). As shown in Fig. 2, our methodology involved quadratic regression. This analysis revealed a strong correlation among the physicochemical properties of effective drugs and specific qualities derived from related neighborhood topological indices.

- The first step involved identifying the top arrhythmia medications from existing studies and converting their molecular structures into graphs using chemical graph theory.
- We analyzed the structures by classifying their edges according to the number of bonds on their starting and ending atoms, and then tallied the frequency of each category.
- To save time on manual computations, a Python program was developed to automate the calculation of topological indices and entropies.
- For more precise predictions of physicochemical properties, regression models were constructed using the indices. We used an Anaconda environment to guarantee consistent results and enhance computational speed.
- We visualize the models on the basis of *R*, $R^2$ and *P* values and drew the scatter plots.
- By using MCDM (TOPSIS and SAW) we ranked anti arrhythmia drugs.

## Results and discussion

In this section, we will correlate neighborhood degree sum-based topological indices with physicochemical properties of ten anti-arrhythmia drugs to demonstrate the potential of these indices in predicting drug behavior and aiding in drug design. We will apply TOPSIS and SAW multi-criteria decision-making approaches, combined with the entropy method for weight allocation, to rank the drugs, highlighting their potential effectiveness based on the analyzed properties.

Let $\varGamma$ is the chemical graph of Metoprolol, so the neighborhood M-polynomial is:

2 $$\begin{aligned} NM(\varGamma ;x,y) = x^2y^3 + 3x^3y^4+x^3y^5 + 2x^4y^5 + 6x^5y^5 + 6x^5y^6 \end{aligned}$$

Let $\varGamma$ is the chemical graph of Propranolol, so the neighborhood M-polynomial is:

3 $$\begin{aligned} NM(\varGamma ,x,y) = 2x^3y^4 + x^4y^4 + 5x^4y^5 + x^3y^5 + 4x^5y^5 + 4x^5y^7 + x^5y^8 + 2x^7y^8 \end{aligned}$$

Let $\varGamma$ is the chemical graph of Atenolol, then the neighborhood M-polynomial is:

4 $$\begin{aligned} NM(\varGamma ,x,y)= x^2y^3 + 2x^3y^4 + 2x^3y^5 + x^4y^6 + 5x^5y^5 + 5x^5y^6 + x^6y^6 \end{aligned}$$

Let $\varGamma$ is the chemical graph of Bisoprolol, so the neighborhood M-polynomial is:

5 $$\begin{aligned} NM(\varGamma ,x,y) = 4x^3y^4 + x^3y^5 + 2x^4y^4 + 4x^4y^5 + 6x^5y^5 + 6x^5y^6 \end{aligned}$$

Let $\varGamma$ is the chemical graph of Sotalol, so the neighborhood M-polynomial is:

6 $$\begin{aligned} NM(\varGamma ,x,y) = 2x^3y^4 + x^3y^5 + 4x^4y^5 + 4x^5y^5 + 2x^5y^6+ 4x^5y^7 + x^6y^7 \end{aligned}$$

Let $\varGamma$ is the chemical graph of Amiodarone, so the neighborhood M-polynomial is:

7 $$\begin{aligned} NM(\varGamma ,x,y)&= x^2y^3 +2 x^2y^4 + x^3y^4 + 2x^3y^6 + x^3y^7 +x^4y^4+5x^4y^5+2x^4y^6+x^5y^6\\ & \quad+2x^5y^7+2x^5y^8+2x^6y^6+4x^6y^7+2x^6y^8+x^7y^7+x^7y^8+2x^7y^9 +x^8y^9\end{aligned}$$

Let $\varGamma$ is the chemical graph of Carvedilol, so the neighborhood M-polynomial is;

8 $$\begin{aligned} NM(\varGamma ,x,y)&= x^2y^4 + 4x^4y^4 + 8x^4y^5 + x^4y^7 + x^5y^3 + 7x^5y^7 +2 x^5y^5 + x^5y^8 + 2x^6y^7 + x^7y^7 \\ & \quad+ x^7y^8 + 2x^7y^9 \end{aligned}$$

Let $\varGamma$ is the chemical graph of Flecainide, so the neighborhood M-polynomial is:

9 $$\begin{aligned} NM(\varGamma ,x,y) = x^3y^6 + 2x^4y^4 + 8x^4y^5 + 5x^5y^5 + 7x^5y^6 + 2x^5y^7 + x^6y^6 + 2x^6y^8 + x^7y^8 \end{aligned}$$

Let $\varGamma$ is the chemical graph of Timolol, so the neighborhood M-polynomial is:

10 $$\begin{aligned} NM(\varGamma ,x,y) = x^3y^5 + 2x^4y^4 + 7x^4y^5 + 3x^5y^5 + 2x^5y^6 + 4x^5y^7 + x^5y^8 + 2x^7y^8 \end{aligned}$$

Let $\varGamma$ is the chemical graph of Propafenone, so the neighborhood M-polynomial is:

11 $$\begin{aligned} NM(\varGamma ,x,y) = x^2y^3 + x^3y^4 + x^3y^5 + x^3y^6 + 4x^4y^4 + 5x^4y^5 + 4x^5y^5 + 4x^5y^6 + 2x^5y^7 + x^5y^8 + x^6y^8+x^7y^8 \end{aligned}$$

By using Eqs. (2)–(11) and Table 1 we get Table 2. Where as, Table 3 shows the physicochemical properties of anti arrhythmia drugs sourced from Chemspider.

**Table 2:** Topological indices of anti-arrhythmia drugs.

Drugs	$M_1(\varGamma )$	$M_2(\varGamma )$	$NM_1(\varGamma )$	$NM_2(\varGamma )$	$NSS(\varGamma )$	$NH(\varGamma )$
Metoprolol	84	89	178	427	28.77	4.24
Atenolol	86	91	162	395	25.9	3.75
Bisoprolol	102	107	214	505	34.79	5.07
Propranolol	92	103	206	547	31.63	4.07
Sotalol	86	92	181	461	28.21	3.69
Amiodarone	156	184	368	1074	53.72	6.46
Carvedilol	154	180	360	1029	53.58	6.41
Flecainide	140	153	303	803	46.42	5.69
Propafenone	116	128	256	649	40.18	5.56
Timolol	104	115	230	614	35.13	4.36

**Table 3:** Physicochemical properties of anti-arrhythmia drugs.

Drugs	D	BP	FP	BCF	KOC	*P*	MV
Metoprolol	1.00	398.60	194.90	1.00	1.00	30.60	258.70
Atenolol	1.10	508.00	261.10	1.00	1.00	29.40	236.70
Bisoprolol	1.00	445.00	222.90	1.00	1.00	36.70	315.00
Propranolol	1.10	434.90	216.80	1.25	1.00	31.30	237.20
Sotalol	1.20	443.30	221.90	1.00	1.00	28.60	219.70
Amiodarone	1.60	635.10	337.90	214.87	223.42	57.20	408.20
Carvedilol	1.30	655.20	350.10	1.51	8.15	47.40	325.10
Flecainide	1.30	434.90	216.80	1.00	1.07	34.80	322.10
Propafenone	1.10	519.60	268.00	1.00	1.61	39.80	311.40
Timolol	1.20	487.20	248.50	1.00	1.00	32.60	258.50

## QSPR analysis of anti-arrhythmia drugs

In this study, we apply quadratic QSPR (Quantitative Structure-Property Relationship) models to understand the connections between these measurements, aiming to identify the most effective treatments for arrhythmia by studying their molecular properties. Table 3 lists the physicochemical properties of the arrhythmia drugs under investigation sourced from Chemspider. This work draws inspiration from prior studies on anti-cancer drugs, and QSPR analysis of various topological indices across different molecular architectures. This research aims to explore the effectiveness of topological indices (TIs) in replicating QSPR properties and their potential in managing arrhythmia treatment. A quadratic regression model is a statistical method used to define the relationship between a dependent variable and one or more independent variables. The general form of a quadratic regression equation is typically expressed as:

$$\begin{aligned} P=A_\circ +B_\circ (TI)+C_\circ (TI)^2 \end{aligned}$$

where $A_\circ , B_\circ , C_\circ$ are constants. We are using a quadratic regression model because it offers greater flexibility in linking topological indices (independent variables), to chemical properties P (response variables). This allows us to thoroughly examine the relationships between these indices and the compounds’ characteristics. Our aim is to pinpoint the strongest correlations, uncovering patterns that help to anticipate or interpret chemical behaviors. Statistical details for the quadratic QSPR model, including various topological indices, are presented in Tables 4 through 9. Additionally, Table 10 illustrates the correlation coefficients between these indices and different properties. All computations were performed using (SPSS Statistics 27.0.1.0, (https://www.ibm.com/support/pages/downloading-ibm-spss-statistics-27010)). We specifically selected the topological indices $M_1(\varGamma )$, $M_2(\varGamma )$, $NM_1(\varGamma )$, $NM_2(\varGamma )$, $NH(\varGamma )$, $NSS(\varGamma )$ because of their strong correlations with the physicochemical properties of potential arrhythmia drugs. We confirmed these correlations using a quadratic regression model in SPSS, which showed these indices were not chosen arbitrarily but for their significant relationship with drug properties. This deliberate selection enabled more effective ranking of the drugs and informed decision-making regarding their use in arrhythmia treatment.

The following sections will detail the quadratic regression model’s findings, discussing the various physicochemical properties of these potential drugs in relation to the topological indices for arrhythmia disease, all computed by using SPSS software.

### Regression models for $M_1(\varGamma )$

$$\begin{aligned} D&= 2.0622 - 0.0208 M_1(\varGamma ) + 0.0001 M_1^2(\varGamma )\\ BP&= 603.7442 - 3.7566 M_1(\varGamma ) + 0.0229 M_1^2(\varGamma ) \\ FP&= 479.0312 - 5.3411 M_1(\varGamma ) + 0.0281 M_1^2 (\varGamma )\\ BCF&= 568.9568 - 10.9117 M_1(\varGamma ) + 0.0509 M_1^2(\varGamma ) \\ KOC&= 600.5683 - 11.5314 M_1(\varGamma ) + 0.0539 M_1^2 (\varGamma )\\ P&= 34.9526 - 0.2498 M_1 (\varGamma )+ 0.0023 M_1^2 (\varGamma )\\ MV&= 7.7104 + 3.2116 M_1(\varGamma ) - 0.0058 M_1^2(\varGamma ) \\ \end{aligned}$$

### Regression models for $M_2(\varGamma )$

$$\begin{aligned} D&= 1.5423 - 0.0098 M_2 (\varGamma )+ 0.0001 M_2^2 (\varGamma )\\ BP&= 565.0199 - 2.608 M_2(\varGamma ) + 0.0148 M_2^2 (\varGamma )\\ FP&= 413.4675 - 3.6374 M_2(\varGamma ) + 0.0176 M_2^2(\varGamma ) \\ BCF&= 416.8889 - 7.2842 M_2(\varGamma ) + 0.0307 M_2^2(\varGamma ) \\ KOC&= 438.5572 - 7.6750 M_2(\varGamma ) + 0.0325 M_2^2 (\varGamma )\\ P&= 32.1071 - 0.1474 M_2(\varGamma ) + 0.0014 M_2^2(\varGamma ) \\ MV&= 77.6323 + 2.1015 M_2(\varGamma ) - 0.0023 M_2^2(\varGamma ) \\ \end{aligned}$$

### Regression models for $NM_1(\varGamma )$

$$\begin{aligned} D&= 1.5641 - 0.0051 NM_1(\varGamma ) + 0.0000 NM_1^2(\varGamma ) \\ BP&= 669.01 - 2.1284 NM_1(\varGamma ) + 0.0052 NM_1^2 (\varGamma )\\ FP&= 451.8066 - 2.1361 NM_1 (\varGamma )+ 0.0050 NM_1^2(\varGamma ) \\ BCF&= 330.5274 - 3.0644 NM_1(\varGamma ) + 0.0066 NM_1^2(\varGamma ) \\ KOC&= 348.1879 - 3.1703 NM_1(\varGamma ) + 0.0070 NM_1^2(\varGamma ) \\ P&= 32.4871 - 0.0750 NM_1(\varGamma ) + 0.0004 NM_1^2(\varGamma ) \\ MV&= 112.3116 + 0.7657 NM_1 (\varGamma )- 0.0002 NM_1^2(\varGamma ) \\ \end{aligned}$$

### Regression models for $NM_2(\varGamma )$

$$\begin{aligned} D&= 1.1889 - 0.0007 NM_2(\varGamma ) + 0.0000 NM_2^2(\varGamma ) \\ BP&= 532.0131 -0.3437 NM_2(\varGamma ) + 0.0004NM_2^2 (\varGamma )\\ FP&= 339.3478 -0.4302 NM_2(\varGamma ) + 0.0004 NM_2^2(\varGamma ) \\ BCF&=225.1993 -0.7842NM_2 (\varGamma )+ 0.0006 NM_2^2(\varGamma ) \\ KOC&=235.3215 -0.8216 NM_2(\varGamma ) + 0.0007 NM_2^2(\varGamma ) \\ P&= 30.9501 - 0.0155 NM_2 (\varGamma )+ 0.0000 NM_2^2(\varGamma ) \\ MV&= 177.9734 + 0.1439 NM_2(\varGamma ) - 0.0000 NM_2^2(\varGamma ) \\ \end{aligned}$$

### Regression models for $NSS(\varGamma )$

$$\begin{aligned} D&=1.5038- 0.0306NSS(\varGamma )+ 0.0005NSS^2 (\varGamma )\\ BP&=826.218-22.7316NSS(\varGamma )+0.3414NSS^2 (\varGamma )\\ FP&= 524.279318.1217NSS(\varGamma )+ 0.2725NSS^2(\varGamma ) \\ BCF&=247.5362 -15.3273NSS(\varGamma )+ 0.2329NSS^2(\varGamma ) \\ KOC&=267.1751-16.5314NSS(\varGamma )+ 0.2510NSS^2(\varGamma ) \\ P&= 24.2235- 0.1429 NSS(\varGamma ) + 0.0120 NSS^2(\varGamma ) \\ MV&=-10.7559 + 10.8872 NSS(\varGamma )- 0.0724NSS^2(\varGamma ) \\ \end{aligned}$$

### Regression models for $NH(\varGamma )$

$$\begin{aligned} D&=3.1426- 0.9249NH(\varGamma )+ 0.1032NH^2(\varGamma ) \\ BP&=1395.9975-410.6739NH(\varGamma )+44.2795NH^2(\varGamma ) \\ FP&=1020.7927-345.6808NH(\varGamma )+ 37.2199NH^2(\varGamma ) \\ BCF&=789.8855 -345.4214NH(\varGamma )+37.1511NH^2(\varGamma ) \\ KOC&=837.322-366.4765NH(\varGamma )+ 39.4514NH^2(\varGamma ) \\ P&= 52.5421- 14.6256 NH (\varGamma )+ 2.2526 NH^2(\varGamma ) \\ MV&=-121.4233 + 112.4479 NH(\varGamma ) - 5.6469NH^2 (\varGamma )\\ \end{aligned}$$

**Table 4:** Regression results for $M_1(\varGamma )$.

Property	N	A	B	C	R	R$\phantom{0}^2$	F	RMSE	*P*
D	10	2.0622	−0.0208	0.0001	0.8539	0.7292	9.423	0.0925	0.01
BP	10	603.7442	−3.7566	0.0229	0.6792	0.4612	2.9978	51.48	0.11
FP	10	479.0312	−5.3411	0.0281	0.8008	0.6412	6.256	29.86	0.02
BCF	10	568.9568	−10.9117	0.0509	0.6609	0.4367	2.713	0.275	0.13
KOC	10	600.5683	−11.5314	0.0539	0.6798	0.4621	3.0006	48.77	0.11
P	10	34.9526	−0.2498	0.0023	0.9175	0.8419	18.63	3.45	0.001
MV	10	7.7104	3.2116	−0.0058	0.8794	0.7733	10.9397	26.35	0.005

**Table 5:** Regression results for $M_2(\varGamma )$.

Property	N	A	B	C	R	R$\phantom{0}^2$	F	RMSE	*P*
D	10	1.54	−0.009	0.0001	0.8554	0.7316	9.54	0.921	0.01
BP	10	565.01	−2.608	0.0148	0.7137	0.5093	3.63	49.73	0.08
FP	10	413.46	−3.637	0.0176	0.8462	0.7161	8.82	26.56	0.01
BCF	10	416.88	−7.284	0.0307	0.7028	0.4940	3.42	45.62	0.09
KOC	10	438.55	−7.675	0.0325	0.7220	0.5213	3.81	72.20	0.07
P	10	32.10	−0.147	0.0014	0.9342	0.8727	23.99	3.81	0.007
MV	10	77.63	2.101	−0.0023	0.8657	0.7495	10.47	23.99	0.007

**Table 6:** Regression results for $NM_1(\varGamma )$.

Property	N	A	B	C	R	R$\phantom{0}^2$	F	RMSE	*P*
D	10	1.5641	−0.0051	0.0000	0.8545	0.7302	9.47	0.0923	0.01
BP	10	669.01	−2.128	0.0052	0.7257	0.5266	3.89	48.26	0.07
FP	10	451.80	−2.13	0.0050	0.8622	0.7434	10.13	25.25	0.08
BCF	10	330.52	−3.00	0.0066	0.6976	0.4876	3.32	45.95	0.09
KOC	10	348.18	−3.17	0.0070	0.7170	0.5141	3.70	46.35	0.08
P	10	32.48	−0.075	0.0004	0.9391	0.8820	25.66	3.012	0.0006
MV	10	112.31	−0.766	−0.0002	0.8681	0.7535	10.70	27.47	0.07

**Table 7:** Regression results for $NM_2(\varGamma )$.

Property	N	A	B	C	R	R$\phantom{0}^2$	F	RMSE	*P*
D	10	1.8	−0.0007	0.0000	0.8615	0.7421	10.07	0.0903	0.0008
BP	10	523.01	−0.343	0.0004	0.7210	0.5199	3.78	48.60	0.07
FP	10	339.3	−0.430	0.0004	0.8695	0.7561	10.84	24.62	0.007
BCF	10	225.19	−0.784	0.0006	0.7398	0.5473	4.23	43.15	0.06
KOC	10	235.32	−0.821	0.0007	0.7583	0.575	4.73	43.35	0.05
P	10	30.95	−0.015	0.0000	0.9367	0.8773	25.03	3.04	0.0006
MV	10	177.97	0.1439	0.0000	0.8373	0.7010	8.21	30.26	0.01

**Table 8:** Regression results for $NSS(\varGamma )$.

Property	N	A	B	C	R	R$\phantom{0}^2$	F	RMSE	*P*
D	10	1.50	−0.0306	0.0005	0.8032	0.6451	6.36	0.1059	0.02
BP	10	826.21	−22.73	0.3414	0.7985	0.6377	6.15	42.22	0.02
FP	10	524.28	−18.12	0.2725	0.9001	0.8102	14.94	21.72	0.003
BCF	10	247.53	−15.32	0.232	0.6198	0.3841	2.18	50.33	0.18
KOC	10	267.17	−16.53	0.251	0.6422	0.4124	2.46	50.97	0.15
P	10	24.22	−0.142	0.012	0.9404	0.88844	26.77	2.97	0.0005
MV	10	−10.7559	10.8872	−0.0724	0.8767	0.7686	11.62	26.62	0.006

**Table 9:** Regression results for $NH(\varGamma )$.

Property	N	A	B	C	R	R$\phantom{0}^2$	F	RMSE	*P*
D	10	3.24	−0.9249	0.1032	0.8266	0.6823	7.55	0.10	0.02
BP	10	1395.99	−410.67	44.28	0.7422	0.5509	4.29	47.01	0.06
FP	10	1020.79	−345.68	37.22	0.8703	0.7574	10.93	24.56	0.007
BCF	10	789.88	−345.42	37.15	0.6708	0.4499	2.86	47.57	0.12
KOC	10	837.32	−366.47	39.45	0.6908	0.4772	3.19	48.08	0.10
P	10	52.54	−14.62	2.25	0.9441	0.8913	28.69	2.86	0.0004
MV	10	−121.42	112.44	−5.64	0.9240	0.8537	20.42	0.81	0.001

We use things like $R^2$, and Adjusted $R^2$ to see how reliable our prediction formulas are, and they are defined as: $R^2 = 1 - \frac{\sum _{i=1}^{N} (w_i - \hat{w}_i)^2}{\sum _{i=1}^{N} (w_i - \bar{w})^2}$,

$$\text {Adj-R}^2 = 1 - \frac{(1 - R^2)(N - 1)}{N - p - 1}$$.

Where $w_i$ is the actual value of the dependent variable, $\hat{w}_i$ is predicted value of the dependent variable, $\bar{w}$ is mean of the actual values of dependent variable and *p* represents several predictors used in the regression model. N represents the number of data points we used to create the prediction formulas. changeR-squared$R^2$ tells us how well the formulas fit the data. $R^2$ and Adj-$R^2$ close to 1 indicate a good regression model. To get more information we refer to^32^.

As shown in Table 4, the $M_1(\varGamma )$ index’s regression models and statistical parameters highlight its strong predictive capability for Density, Molar Volume, and Flash Point, with high R values (0.8539, 0.8794, and 0.8008). However, its predictive power for Boiling Point, BCF, and KOC is only average, given their moderate R values and high *P*-values. Models for Polarizability demonstrate weak prediction with lower *R* values. From Table 5, it is clear that regression models for density, flash point, polarizability and molar volume have high correlation coefficient values ($R\ge 0.8$), low ($P\le 0.05$) and all these models have high F-statistics values which show models are statistically efficient to predict values of physicochemical properties of anti arrhythmia drugs. Table 6, shows that polarizability has high R-value (0.9391) that is $NM_1(\varGamma )$ is strong predictor for polarizability. $NM_1(\varGamma )$ show moderate correlation for density, flash point, and molar volume with R-values (0.8545, 0.8622, 0.8681). Table 7, shows that the regression models for polarizability, density, flash point and molar volume are strong predictors, evidenced by their high R-values (0.9367, 0.8615, 0.8695 and0.8373 respectively) and low *P*-values$(\le 0.05)$. This suggests that models like BCF, KOC and BP, with their lower values are good predictors for these properties.

Tables 8 and 9, show that the regression models for polarizability, density, flash point and molar volume are strong predictors, evidenced by their high R-values (0.9404, 0.8032, 0.9001 and 0.8767), (0.9441, 0.8266, 0.8703 and 0.9240) respectively, and low *P*-values$(\le 0.05)$. This suggests that models like BP, with their lower values, are moderate predictors for boiling point. For BCF and KOC, the model shows a much lower predictive capability, which is reflected in their respective R-values, indicating that $NSS(\varGamma )$ and $NH(\varGamma )$ cannot predict these properties with high accuracy. Table 10, illustrates the correlation coefficients between topological indices and physical and chemical properties of anti arrhythmia drugs.

**Table 10:** Correlation coefficients between topological indices and physicochemical properties.

Topological indices	D	BP	FP	BCF	KOC	*P*	MV
$M_1(\varGamma )$	0.8539	0.6792	0.8008	0.6609	0.6798	0.9175	0.8794
$M_2(\varGamma )$	${\textbf {0.8554}}$	0.7137	0.8462	0.7028	0.7222	0.9342	0.8657
$NM_1(\varGamma )$	0.8545	0.7257	0.8622	0.6976	0.7170	0.9391	0.8681
$NM_2(\varGamma )$	0.8615	0.7210	0.8695	${\textbf {0.7398}}$	${\textbf {0.7583}}$	0.9367	0.8373
$NSS(\varGamma )$	0.8032	${\textbf {0.7985}}$	${\textbf {0.9001}}$	0.6198	0.6422	0.9404	${\textbf {0.9767}}$
$NH(\varGamma )$	0.8266	0.7422	0.8703	0.6708	0.6908	${\textbf {0.9441}}$	0.9240

Siginificance values are in bold.

**Fig. 3:**
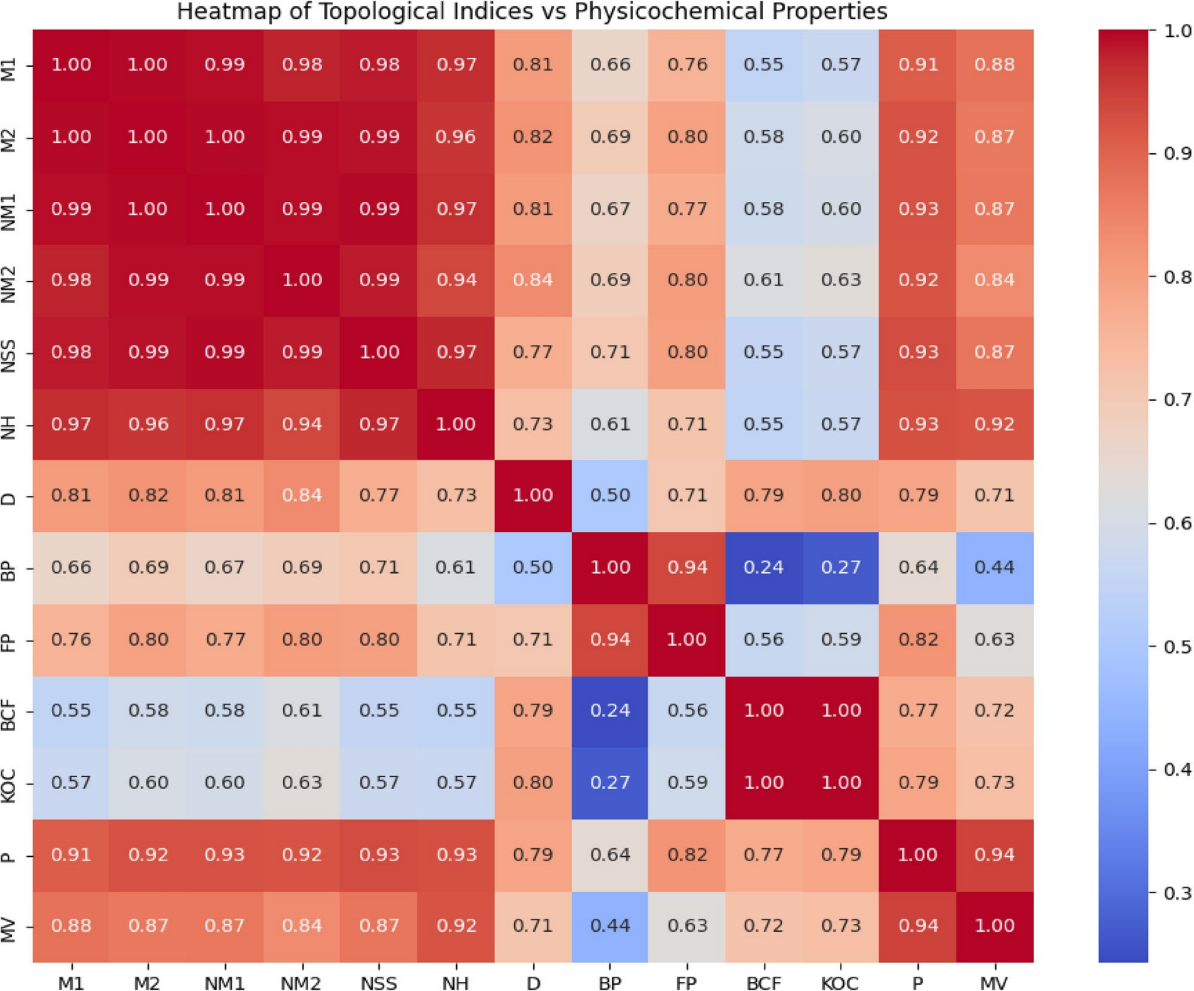
Heatmap of topological indices vs physicochemical properties.

Figure 3, visualize the correlation between topological indices and physicochemical properties which offers a powerful graphical representation for understanding intricate relationships in chemical systems. Each cell within the heatmap displays the correlation coefficient between a specific topological index derived from molecular structures of anti arrhythmia drugs and a particular physicochemical property (like boiling point, density). This visualization allows researchers to quickly identify which structural descriptors are most strongly correlated, positively or negatively, with various molecular characteristics, aiding in property prediction and rational drug design.

**Fig. 4:**
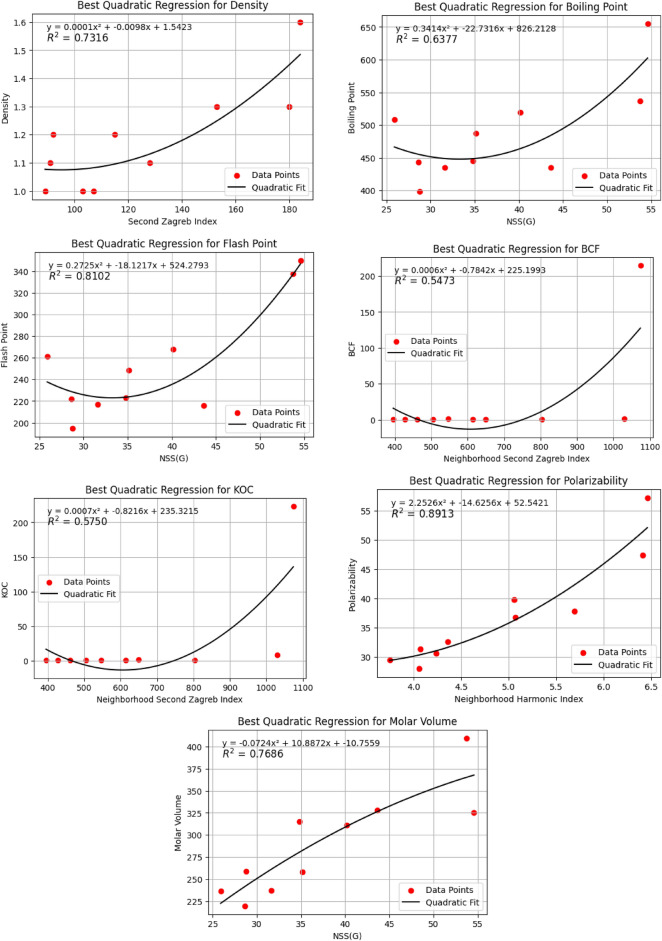
Plots of quadratic regression models that predict optimal physicochemical properties using degree-based topological indices.

The Figure 4, presents the optimal quadratic fits for *D* by $M_2(\varGamma )$, *BP* by $NSS(\varGamma )$, *FP* by $NSS(\varGamma )$, *BCF* by $NM_2(\varGamma )$, *KOC* by $NM_2(\varGamma )$, *P* by $NH(\varGamma )$, and *MV* by $NSS(\varGamma )$. The graphs have a quadratic fit with high $R^2$ values that represents the data fairly accurate. The high $R^2$ value (0.8913) indicate that the quadratically related relationship between $NH(\varGamma )$ and *P* is very exact in predicting the data.

## Application of multi-criteria decision making to drug analysis

Multi-Criteria Decision Making (MCDM) helps choose the best option when several good choices exist instead of one perfect answer. This research uses specific types of mathematical descriptors, called degree based and neighborhood degree-based topological indices, to analyze drugs that treat arrhythmia. By performing QSPR (Quantitative Structure-Property Relationship) analysis, the study reveals a strong link between these indices and the physical and chemical characteristics of arrhythmia medications like Metoprolol, Atenolol, Amiodarone, Bisoprolol, Propranolol, Sotalol, Carvedilol, Flecainide, Propafenone and Timolol.

This research investigates the chemical properties of substances by employing various topological indices, including the Zagreb, neighborhood harmonic, neighborhood Zagreb, and neighborhood Shilpa-Shanmukha indices. To efficiently pinpoint both the best and worst possible outcomes, a weighted evaluation of these indices will be performed using a decision-making technique like TOPSIS and SAW.

This study also assesses how accurately molecular compounds are described using mathematical techniques. It combines Multi-Criteria Decision Making (MCDM), a method dating back to the 1980s^33^, with the entropy method to assign weights to various topological indices. The entropy method ensures an impartial assessment of each index’s relevance in predicting drug properties by assigning weights according to data variability. This means that structural features of a drug that show more diversity across compounds will be given greater importance. This systematic approach helps eliminate personal biases, leading to more precise molecular evaluations. The weights are allocated using the formula:

$$\begin{aligned} \sum _{j=1}^{k}w_{j}=1 \end{aligned}$$

Effectiveness of a drug hinges on its physicochemical properties. To identify the most and least desirable characteristics, it’s crucial to examine factors like density, molar volume, and boiling point, flash point, KOC and BFC. For example, a lower density generally leads to better solubility, while a lower melting point makes a drug easier to use. Molar volume plays a role in how a drug crystallizes, with most effective medications typically weighing less than 1000 g/mol. A higher boiling point is beneficial for storage, and the complexity of a drug can impact both how it is administered and its cost.

In Table 13, using the entropy method, we objectively assigned weights to each drug property. This data-driven approach accurately assesses drug characteristics by determining each property’s weight based on its ability to differentiate between drugs, effectively removing any subjective bias.

### Ranking of drugs using TOPSIS

We look at each property on its own, then figure out the best choices by seeing how closely they match what we ideally want. Table 11 presents n criteria such as Zagreb indices and harmonic index, and m alternatives i.e, drug structures. To determine the best option, appropriate attribute weights are assigned.

*Step 1* Construct an evaluation matrix $(r_{ij})_{m\times n}$ representing where each metric connects with each available choice.

*Step 2* Systematize this matrix to get a normalized version $N=(n_{kj})_ {m \times n}$, Table 12.

$$n_{kj} = \frac{r_{kj}}{\sqrt{\sum _{k=1}^{m} r_{kj}^2}}, \quad \forall k = 1,2,3,\dots ,m \text { and} j = 1,2,3,\dots ,n.$$

*Step 3* Let’s break down how we get to our weighted normalized decision matrix, which we are calling $Y_{ij}$ and you can find in Table 14. We created it by applying the weights that were given to us in a separate Table 13. We will do this by using a specific formula

$$\begin{aligned} \sum _{j=1}^{k}w_{j}=1 \end{aligned}$$

The normalized value is $y_{kj} = w_j^* \cdot n_{kj} \quad \forall j = 1, 2, 3,..., k$, where $\sum _{k=1}^n w_k^* = 1$

*Step 4* We are going to determine the best-case scenario ( positive ideal) and the worst-case scenario (negative ideal) for each of our evaluation categories, Table 15. We are going to compare each option to the absolute best possible option (the “ideal” one) and see how much they differ, which is described as, $Q^+ = \{y_k^+, ..., y_j^+\} = \left( \max _{j \in J} \text { or } \min _{j \in J} Y_{kj} \right) Q^- = \{y_k^-, ..., y_j^-\} = \left( \max _{j \in J} \text { or } \min _{j \in J} Y_{kj} \right)$

*Step 5* We measure this difference using a method called Euclidean distance, which calculates the “straight-line” distance in multiple dimensions. This gives us a clear number showing how far apart they are. The optimal solution stands apart from the other options because. $L_{+i} = \sqrt{\sum _{j=1}^{n} (Y_{ij} - Q_{+j})} L_{-i} = \sqrt{\sum _{j=1}^{n} (Y_{ij} - Q_{-j})}$

**Table 11:** Topological indices(attributes) and drugs(alternatives).

Drugs	$M_1(\varGamma )$	$M_2(\varGamma )$	$NM_1(\varGamma )$	$NM_2(\varGamma )$	$NSS(\varGamma )$	$NH(\varGamma )$
Metoprolol	84	89	178	427	28.77	4.24
Atenolol	86	91	162	395	25.9	3.75
Bisoprolol	102	107	214	505	34.79	5.07
Propranolol	92	103	206	547	31.63	4.07
Sotalol	86	92	181	461	28.21	3.69
Amiodarone	156	184	368	1074	53.72	6.46
Carvedilol	154	180	360	1029	53.58	6.41
Flecainide	140	153	303	803	46.42	5.69
Propafenone	116	128	256	649	40.18	5.56
Timolol	104	115	230	614	35.13	4.36

**Table 12:** Decision matrix.

Drugs	${M_1(\varGamma )}$	${M_2(\varGamma )}$	${NM_1(\varGamma )}$	${NM_2(\varGamma )}$	${NSS(\varGamma )}$	${NH(\varGamma )}$
Metoprolol	0.230644	0.218443	0.220074	0.195706	0.233787	0.267839
Atenolol	0.236135	0.223352	0.200292	0.181040	0.210465	0.236886
Bisoprolol	0.280068	0.262623	0.264583	0.231456	0.282706	0.320269
Propranolol	0.252610	0.252805	0.254692	0.250706	0.257028	0.257100
Sotalol	0.236135	0.225806	0.223783	0.211289	0.232487	0.256468
Amiodarone	0.428339	0.451613	0.454984	0.492245	0.436533	0.408075
Carvedilol	0.422847	0.441795	0.445093	0.471620	0.443521	0.404916
Flecainide	0.384407	0.375526	0.374620	0.368038	0.354459	0.359434
Propafenone	0.318508	0.314166	0.316510	0.297455	0.326506	0.319638
Timolol	0.285559	0.282258	0.284365	0.281414	0.285469	0.275419

**Table 13:** Weight allocation using entropy method.

Topological Index	$M_1(\varGamma )$	$M_2(\varGamma )$	$NM_1(\varGamma )$	$NM_2(\varGamma )$	$NSS(\varGamma )$	$NH(\varGamma )$
Weights	0.170372	0.166171	0.164530	0.155294	0.168411	0.175222

**Fig. 5:**
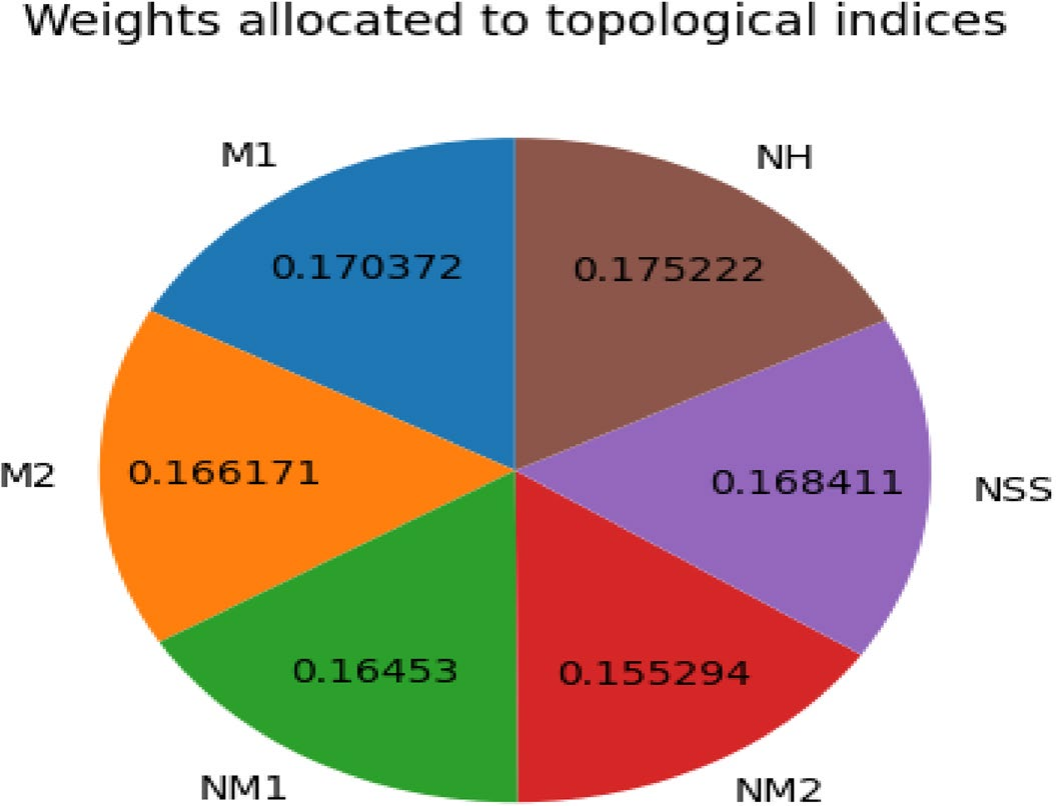
Weights allocated to topological indices.

**Table 14:** Weighted decision matrix.

Drugs	$M_1(\varGamma )$	$M_2(\varGamma )$	$NM_1(\varGamma )$	$NM_2(\varGamma )$	$NSS(\varGamma )$	$NH(\varGamma )$
Metoprolol	0.039295	0.036299	0.036209	0.030392	0.039372	0.046931
Atenolol	0.040231	0.037115	0.032954	0.028114	0.035444	0.041508
Bisoprolol	0.047716	0.043640	0.043532	0.035944	0.047611	0.056118
Propranolol	0.043038	0.042009	0.041904	0.038933	0.043286	0.045050
Sotalol	0.040231	0.037522	0.036819	0.032812	0.039153	0.044939
Amiodarone	0.072977	0.075045	0.074858	0.076443	0.073517	0.071504
Carvedilol	0.072041	0.073414	0.073231	0.073240	0.074693	0.070950
Flecainide	0.065492	0.062402	0.061636	0.057154	0.059694	0.062981
Propafenone	0.054265	0.052205	0.052075	0.046193	0.054987	0.056008
Timolol	0.048651	0.046903	0.046787	0.043702	0.048076	0.048259

**Table 15:** Calculation of ideal best and ideal worst.

	$M_1(\varGamma )$	$M_2(\varGamma )$	$NM_1(\varGamma )$	$NM_2(\varGamma )$	${NSS(\varGamma )}$	${NH(\varGamma )}$
Ideal Best ($P^+$)	0.072977	0.075045	0.074858	0.076443	0.074693	0.071504
Ideal Worst ($P^-$)	0.039295	0.036299	0.032954	0.028114	0.035444	0.041508

**Table 16:** Drug ranking using TOPSIS.

Drugs	$L^+$	$L^-$	$O'_i$	Rank
Amiodarone	0.001176	0.095272	0.987807	1
Carvedilol	0.004093	0.092281	0.957534	2
Flecainide	0.032563	0.063897	0.662416	3
Propafenone	0.054170	0.041973	0.436569	4
Timolol	0.067026	0.028973	0.301809	5
Bisoprolol	0.072222	0.025681	0.262307	6
Propranolol	0.078530	0.017827	0.185013	7
Sotalol	0.088316	0.008057	0.083599	8
Metoprolol	0.090009	0.007786	0.079616	9
Atenolol	0.095094	0.001242	0.012890	10

Figure 5, depicts the weights allocated to the topological indices. The Table 12 shows a decision matrix contrasting different drugs according to the value of different topological indices, such as $M_1(\varGamma )$, $M_2(\varGamma )$, $NM_1(\varGamma )$, $NM_2(\varGamma )$, $NSS(\varGamma )$, $NH(\varGamma )$. Each drug corresponds to particular numerical scores for these indices. For example, Bisoprolol has scores of 0.280068, 0.262623, and 0.264583 for $M_1(\varGamma )$, $M_2(\varGamma )$, and $NM_1(\varGamma )$, respectively, but Atenolol displays lower scores, such as 0.236135 for $M_1(\varGamma )$. This matrix can not predict if a drug will work, but it does assess how well drugs perform based on their topological characteristics. These characteristics might be related to a drug’s effectiveness in treating conditions like arrhythmia. The scores in the matrix show quantitative differences, which could help in making decisions since specific index scores should correlate with therapeutic success.

Now we compute weights for each neighborhood degree based on topological indices in Table 13, and the weighted decision matrix Table 14.

*Step 6* Determine the relative proximity $B_{i}$ of each alternative answer to the ideal answer Table 15.

$$O_k^\prime = \frac{L_{-k}}{L_{+k} + L_{-k}}, \quad \text {where } 0< O_k^\prime < 1, \quad k = 1, 2, 3,..., n.$$

$$\text {It is clear that } O_k^\prime = 1 \text { if } Q_k = Q_+ \text { and } O_k^\prime = 0 \text { if } Q_k = Q_-.$$

*Step 7* This step involves ordering the reference answers from most similar to the ideal answer to least similar, based on the calculated proximity values $O_k^\prime$. The references are ranked in descending order of $O_k^\prime$ Table16.

Our study establishes what constitutes the best and worst choices by examining specific properties that influence how effective medicine is. The ideal best picks comprise high boiling point, polarizability, BCF and KOC. High boiling point can improve drug stability. This means the drug is less likely to break down or degrade at typical storage, A stable drug guarantees that patients get the correct amount of the active ingredient, and the medicine remains effective for its entire shelf life. A drug’s polarizability helps it interact well with different biological molecules and environments, which directly affects its ADME profile (how it’s absorbed, distributed, metabolized, and excreted). Higher value of BCF could reduce the required dosage frequency and potentially enhance therapeutic effect. A medicine with a high value of KOC means that the drug is less likely to leach into water sources, which is a significant environmental and public health benefit.

On the other hand, the ideal worst picks comprise lower values of boiling point, density, and molar volume. Lower boiling point make the compound unstable. A drug which has a very small molecular volume, it might be processed and eliminated from the body quickly. This shortens the time the drug stays active, meaning patients would need to take it more often. Such frequent dosing can make it harder for patients to stick to their medication schedule, potentially leading to inconsistent drug levels in their system which make it worst. When deciding on a drug for arrhythmia, we prioritize characteristics that improve how well it works, giving them more importance in our evaluation. On the other hand, we give less weight to properties that might reduce its effectiveness. This organized method helps us more effectively evaluate and select the best drug candidates for disease treatment.

### SAW-based drug ranking

The Simple Additive Weighting (SAW) method, sometimes called the weighted scoring method, is a multi-criteria decision-making tool. It operates by computing a weighted average for each available option. In essence, SAW calculates an overall score for each alternative by multiplying its performance on various criteria by the corresponding importance (weight) of those criteria, and then summing these products. The steps involved in the SAW method compromise ranking procedure are:

*Step 1* Create a conclusion matrix Table 11, list the m alternatives and n attributes, identifying the best and worst values for each attribute.

*Step 2* Determine the weights by using previously established weighted norms. Subsequently, construct a normalized decision matrix Table 17, using the following formulas:

$$m_{ij} = \frac{j_{kj}}{\max (j_{kj})}$$

$$m_{ij} = \frac{\min (j_{kj})}{j_{kj}}$$

where $j_{kj}$ is the original value, $k = 1, 2, 3,..., m$ and $j = 1, 2, 3,..., n$.

*Step 3* Find the value of each replacement by applying the formula. The formula to calculate each substitute’s score is:

$$G_k = \sum _{j=1}^{n} w_j \cdot n_{ij}$$

where $G_k$ is the weighted sum for substitute *i*, $w_j$ is the weight of attribute *j*. $n_{ij}$ is the normalized value of substitute *i* for attribute *j*, and *n* is the total number of attributes Table 18.

Our research is really useful for making new medicines, especially for arrhythmia problems. We use special math tools and computer models to figure out how well different drugs might work, Table 19. This approach can help drug companies identify the most promising drug candidates for early testing, which saves them both time and money. Instead of lab-testing every compound, they can use our method to predict which ones are most likely to be effective. This technique is not just for drugs; it can also be useful for environmental chemical analysis or developing new materials.

**Table 17:** Normalized decision matrix.

Drugs	${M_1(\varGamma )}$	${M_2(\varGamma )}$	${NM_1(\varGamma )}$	${NM_2(\varGamma )}$	${NSS(\varGamma )}$	${NH(\varGamma )}$
Metoprolol	0.538462	0.483696	0.483696	0.397579	0.527116	0.656347
Atenolol	0.551282	0.494565	0.440217	0.367784	0.474533	0.580495
Bisoprolol	0.653846	0.581522	0.581522	0.470205	0.637413	0.784830
Propranolol	0.589744	0.559783	0.559783	0.509311	0.579516	0.630031
Sotalol	0.551282	0.500000	0.491848	0.429236	0.524185	0.628483
Amiodarone	1.000000	1.000000	1.000000	1.000000	0.984243	1.000000
Carvedilol	0.987179	0.978261	0.978261	0.958101	1.000000	0.992260
Flecainide	0.897436	0.831522	0.823370	0.747672	0.799194	0.880805
Propafenone	0.743590	0.695652	0.695652	0.604283	0.736167	0.783282
Timolol	0.666667	0.625000	0.625000	0.571695	0.643642	0.674923

The normalized decision matrix of different drugs according to topological indices in Table 17 represents the standardized value of the different indices (i.e., $M_1(\varGamma )$, $M_2(\varGamma )$, $NM_1(\varGamma )$, $NM_2(\varGamma )$, $NSS(\varGamma )$, and $NH(\varGamma )$) of each drug. The normalization process allows the different indices of all the drugs to be on the same scale, so any comparison of the drugs becomes possible on the same level. For instance, “Amiodarone” has the highest normalized value of 1.000000 in the case of $M_1(\varGamma )$, $M_2(\varGamma )$, $NM_1(\varGamma )$, $NM_2(\varGamma )$, and $NH(\varGamma )$. Conversely, “Atenolol” possesses comparatively lower normalized value across all the indices; it may thus not be able to perform better in accordance with these topological characteristics. Normalization is a crucial step in preparing data because it puts all the drugs on an equal footing. This means we can compare them fairly, no matter how large or small their original data values were.

**Table 18:** Weighted normalized decision matrix.

Drugs	${M_1(\varGamma )}$	${M_2(\varGamma )}$	${NM_1(\varGamma )}$	${NM_2(\varGamma )}$	${NSS(\varGamma )}$	${NH(\varGamma )}$
Metoprolol	0.091739	0.080376	0.079582	0.061742	0.088772	0.115006
Atenolol	0.093923	0.082182	0.072429	0.057115	0.079917	0.101716
Bisoprolol	0.111397	0.096632	0.095678	0.073020	0.107347	0.137519
Propranolol	0.100476	0.093020	0.092101	0.079093	0.097597	0.110395
Sotalol	0.093923	0.083086	0.080924	0.066658	0.088278	0.110124
Amiodarone	0.170372	0.166171	0.164530	0.155294	0.165757	0.175222
Carvedilol	0.168188	0.162559	0.160953	0.148787	0.168411	0.173866
Flecainide	0.152898	0.138175	0.135469	0.116109	0.134593	0.154336
Propafenone	0.126687	0.115597	0.114456	0.093842	0.123979	0.137248
Timolol	0.113581	0.103857	0.102831	0.088781	0.108396	0.118261

Table 18, presents the weighted normalized decision matrix. This updated matrix improves upon the previous one by adjusting the importance of each topological index based on its significance for evaluating the drugs, and then applying these adjusted weights to the normalized values. For example, “Amiodarone” consistently ranks highest across most performance indicators, showing its overall strong effectiveness. This is clear from its high SAW (Simple Additive Weighting) score of 0.997346, confirming it as the top performer, just as expected. “Atenolol” however, ranks last with a comparatively lower SAW value of 0.487281, indicating that it has lower preference when the weighted significance of the indices is used. This weighted system highlights the drugs that perform best on the most important criteria, making it a more precise tool for decision-making.

**Table 19:** Final ranking using SAW.

Drugs	SAW score	Rank
Amiodarone	0.997346	1
Carvedilol	0.982764	2
Flecainide	0.831580	3
Propafenone	0.711808	4
Timolol	0.635708	5
Bisoprolol	0.621594	6
Propranolol	0.572682	7
Sotalol	0.522993	8
Metoprolol	0.517218	9
Atenolol	0.487281	10

The final ranking of the drugs according to their SAW scores from the preceding table is presented in Table 19. From the table, it is clear that ”Amiodarone” with a score of 0.997346 rank highest, followed by Carvedilol, and Flecainide with scores of 0.982764 and 0.831580, respectively. This ranking results from the addition of the normalized and weighted scores of each drug, thereby providing a clear comparison. Other drugs like, Metoprolol and Atenolol rank lower, with the last position held by Atenolol. This ranking aids in the informed decision-making process of the most likely best drug.

## Conclusions

This research investigated the molecular structures and physicochemical properties of ten anti arrhythmia drugs. We used various topological indices, such as Shilpa-Shanmukha Index , harmonic, and Zagreb indices, which are based on the neighborhood degree of a molecule’s atoms. Our findings show strong relationships between these indices and the drugs’ properties. Notably, the neighborhood harmonic index was the most effective in predicting polarizability when using quadratic regression models. Additionally, we employed multi-criteria decision-making methods. The entropy method was applied to allocate weights to each topological index, confirming that criteria with higher information content received greater importance during the MCDM ranking with SAW and TOPSIS. This helped us pinpoint the most promising drug candidates. This study provided a cost effective, rapid method for predicting drugs properties and ranking, which can complement time consuming and costly clinical trials.

Future research could explore distance-based topological indices and more complex polynomial regression models to further improve the accuracy of these predictions.

## Data Availability

The datasets used and/or analyzed during the current study are available from the corresponding author on reasonable request.
